# Optical Genome Mapping: A Promising New Tool to Assess Genomic Complexity in Chronic Lymphocytic Leukemia (CLL)

**DOI:** 10.3390/cancers14143376

**Published:** 2022-07-11

**Authors:** Anna Puiggros, Silvia Ramos-Campoy, Joanna Kamaso, Mireia de la Rosa, Marta Salido, Carme Melero, María Rodríguez-Rivera, Sandrine Bougeon, Rosa Collado, Eva Gimeno, Rocío García-Serra, Sara Alonso, Marco Antonio Moro-García, María Dolores García-Malo, Xavier Calvo, Leonor Arenillas, Ana Ferrer, Tuomo Mantere, Alexander Hoischen, Jacqueline Schoumans, Blanca Espinet

**Affiliations:** 1 Molecular Cytogenetics and Hematological Cytology Laboratories, Pathology Department, Hospital del Mar, 08003 Barcelona, Spain; sramosca10@alumnes.ub.edu (S.R.-C.); jkamaso@imim.es (J.K.); mireia.delarosa.9@gmail.com (M.d.l.R.); msalido@psmar.cat (M.S.); mmelero@psmar.cat (C.M.); mmrodriguezrivera@psmar.cat (M.R.-R.); xcalvo@psmar.cat (X.C.); larenillas@psmar.cat (L.A.); aferrera@psmar.cat (A.F.); 2 Translational Research on Hematological Neoplasms Group, Cancer Research Program, Institut Hospital del Mar d’Investigacions Mèdiques (IMIM), 08003 Barcelona, Spain; 3 Oncogenomic Laboratory, Hematology Service, Lausanne University Hospital, 1011 Lausanne, Switzerland; sandrine.bougeon@chuv.ch (S.B.); jacqueline.schoumans@chuv.ch (J.S.); 4 Department of Hematology, Consorcio Hospital General Universitario, 46014 Valencia, Spain; collado_ros@gva.es (R.C.); garcia_rocser@gva.es (R.G.-S.); 5 Department of Hematology, Hospital del Mar, 08003 Barcelona, Spain; egimeno@psmar.cat; 6 Applied Clinical Research in Hematological Malignances, Cancer Research Program, Institut Hospital del Mar d’Investigacions Mèdiques (IMIM), 08003 Barcelona, Spain; 7 Research Foundation from Hospital General Universitario, 46014 Valencia, Spain; 8 Department of Hematology, Hospital Universitario Central de Asturias, 33011 Oviedo, Spain; saralonsoalvarez@gmail.com; 9 Laboratory Medicine Department, Hospital Universitario Central de Asturias, 33011 Oviedo, Spain; marcomorog@hotmail.com; 10 Department of Hematology, Hospital Universitario Morales Meseguer, 30008 Murcia, Spain; garcia_mdomalo@telefonica.net; 11 Department of Human Genetics, Radboud University Medical Center, 6500 Nijmegen, The Netherlands; tuomo.mantere@oulu.fi (T.M.); alexander.hoischen@radboudumc.nl (A.H.); 12 Laboratory of Cancer Genetics and Tumor Biology, Cancer and Translational Medicine Research Unit and Biocenter Oulu, University of Oulu, 90570 Oulu, Finland; 13 Radboud Center for Infectious Diseases (RCI), Department of Internal Medicine and Radboud Institute for Molecular Life Sciences, Radboud University Medical Center, 6532 Nijmegen, The Netherlands

**Keywords:** chronic lymphocytic leukemia, optical genome mapping, chromosome banding analysis, chromosomal microarrays, genomic complexity, prognosis

## Abstract

**Simple Summary:**

Genome complexity, detected by chromosome banding analysis or chromosomal microarray analysis, is a poor prognostic factor for chronic lymphocytic leukemia (CLL). Herein, we aimed to assess the performance of optical genome mapping (OGM) for the cytogenomic characterization of CLL patients, with a special focus on risk stratification based on genomic complexity. A cohort of 42 patients enriched in complex karyotypes was assessed by OGM, and the results were compared with those obtained from current methods. Moreover, clinical–biological characteristics and time to first treatment were analyzed according to the OGM-defined complexity. Globally, OGM identified 90% of the known alterations and provided novel structural information about these aberrations in 55% of patients. Regarding genomic complexity, OGM allowed us to identify a complex group (≥10 alterations) displaying enrichment of *TP53* abnormalities and poorer evolution. Altogether, we confirmed that OGM is a valuable tool for the cytogenomic assessment and prognostic stratification of CLL patients.

**Abstract:**

Novel treatments in chronic lymphocytic leukemia (CLL) have generated interest regarding the clinical impact of genomic complexity, currently assessed by chromosome banding analysis (CBA) and chromosomal microarray analysis (CMA). Optical genome mapping (OGM), a novel technique based on imaging of long DNA molecules labeled at specific sites, allows the identification of multiple cytogenetic abnormalities in a single test. We aimed to determine whether OGM is a suitable alternative to cytogenomic assessment in CLL, especially focused on genomic complexity. Cytogenomic OGM aberrations from 42 patients were compared with CBA, FISH, and CMA information. Clinical–biological characteristics and time to first treatment (TTFT) were analyzed according to the complexity detected by OGM. Globally, OGM identified 90.3% of the known alterations (279/309). Discordances were mainly found in (peri-)centromeric or telomeric regions or subclonal aberrations (<15–20%). OGM underscored additional abnormalities, providing novel structural information on known aberrations in 55% of patients. Regarding genomic complexity, the number of OGM abnormalities had better accuracy in predicting TTFT than current methods (C-index: 0.696, 0.602, 0.661 by OGM, CBA, and CMA, respectively). A cut-off of ≥10 alterations defined a complex OGM group (C-OGM, *n* = 12), which included 11/14 patients with ≥5 abnormalities by CBA/CMA and one patient with chromothripsis (Kappa index = 0.778; *p* < 0.001). Moreover, C-OGM displayed enrichment of *TP53* abnormalities (58.3% vs. 3.3%, *p* < 0.001) and a significantly shorter TTFT (median: 2 vs. 43 months, *p* = 0.014). OGM is a robust technology for implementation in the routine management of CLL patients, although further studies are required to define standard genomic complexity criteria.

## 1. Introduction

Chronic lymphocytic leukemia (CLL) is characterized by the clonal proliferation and accumulation of abnormal mature B cells, typically CD5 positive, in the blood and lymphoid tissues. This disease is the most common leukemia in Western countries and displays a highly variable clinical course, ranging from very indolent cases to patients with aggressive disease and rapid progression [1]. This clinical heterogeneity has important consequences for clinical follow-up, therapeutic strategies, and patient survival. Because of this, several prognostic and predictive biomarkers have been identified over the years [2]; the most relevant being deletions and/or mutations in *TP53*, located at 17p13, and the mutational status of the variable region of the immunoglobulin heavy chain (IGHV) gene [1]. FISH analysis of the four-probe set included in Dohner’s hierarchical model [targeting deletions on 13q14, 11q22 (*ATM*), 17p13 (*TP53*), and trisomy of chromosome 12] is currently considered the gold standard for cytogenetic assessment in CLL [3]. However, this strategy underestimates the true genetic complexity of the tumor cells, which also predicts a more aggressive clinical course [4,5]. Although the negative impact of complex karyotypes (CK) has been extensively demonstrated in patients treated with standard chemoimmunotherapy regimes [6,7,8,9], its clinical relevance in patients receiving new treatment modalities has not been fully established. While initial data from clinical trials with novel agents suggested an adverse significance of CK [10,11,12,13], recent studies, including extended follow-ups of older trials, pooled analyses, or new drug combinations, reported controversial results regarding its adverse significance [14,15,16,17,18,19,20,21]. Thus, additional analyses are required to clarify the prognostic/predictive impact of genomic complexity in the new treatment era.

To date, most of the outcome analyses of genomic complexity published in CLL have been reported using chromosome banding analysis (CBA) data and defined CK as the presence of three or more chromosomal alterations detected in the same cell clone [22,23]. An increasing number of chromosomal abnormalities in the karyotype has been correlated with a worse clinical evolution of CLL patients, and, notably, the types of aberrations detected also modulates its prognostic impact [8,24,25,26]. Moreover, chromosomal microarray analysis (CMA) has replaced CBA and FISH in some laboratories for the detection of copy number variants (CNVs). Two large retrospective studies from the European Research Initiative on CLL (ERIC) demonstrated that genomic complexity, detected by either CBA or CMA, is an independent adverse prognosticator in CLL, five or more being the number of aberrations that best predicted a worse evolution [9,27]. In addition, our group compared the performance of CBA and CMA to detect genomic complexity in a cohort of 340 patients enriched in CK. Although the prognostic utility of both methods was validated in this study, we demonstrated that risk stratification by CBA and CMA was not fully equivalent due to the intrinsic limitations of each technique [28]. The ERIC group recently published their recommendations, in which CBA still remains the gold standard methodology, capable of providing information on structural and numerical aberrations at a low resolution of the whole genome while also allowing the overview of the clonal landscape and intraclonal hierarchy [29]. It is noteworthy that, while CBA results rely on the in vitro proliferative capacity of the altered clones, CNV profiles can be defined at high resolution by CMA without having to culture samples. In some cases, CMA could reveal multiple CNVs or even chromothripsis events associated with monosomies or unknown cytogenetic elements reported in the karyotype. On the contrary, balanced abnormalities are only detectable by CBA, and subclonal alterations present at low percentages (<20%) could be missed by CMA due to its lower sensitivity [28,29,30,31].

Optical genome mapping (OGM) has emerged as a promising novel technique that could potentially overcome the aforementioned limitations in a single test. OGM relies on high-throughput imaging of long DNA molecules (>250 Kb) fluorescently labeled at a specific 6 bp sequence motif, occurring approximately 15 times per 100 Kb in the human genome. The unique labeling pattern throughout the genome confers the ability to unambiguously identify the genomic location of every imaged molecule, generating a local consensus map that can be compared to a reference genome to detect structural variants (SVs). This analysis is performed via the so-called rare variant pipeline, which specifically addresses mosaic samples and automatically detects both balanced and unbalanced SVs from single molecules, genome-wide, starting at 5 Kb and down to a 1% allele fraction. In addition, genome coverage depth information is also used to address copy number variant (CNV) and whole-chromosome aneuploidy identification [32,33]. Several recent publications have demonstrated OGM’s good performance in the cytogenomic assessment of different hematological malignancies, especially focused on myeloid neoplasms (acute myeloid leukemia and myelodysplastic syndromes) and acute lymphoblastic leukemia cases. In these studies, OGM effectively detected most of the clinically relevant abnormalities reported by standard methods and, in some cases, also provided new cytogenomic information [34,35,36,37]. Conversely, the assessment of genomic complexity by OGM or the global characterization of the cytogenetic abnormalities identified in each patient was beyond the scope of the published studies, which focused on the identification of SVs currently interrogated in diagnostic procedures or that overlapped with genes defined as potentially relevant in leukemogenesis.

In the present study, we aimed to assess the performance of OGM for the cytogenomic characterization of CLL patients in comparison with previous CBA and CMA information, with a special focus on genomic complexity. Moreover, we analyzed the potential use of OGM for the prognostic stratification of CLL patients based on genomic complexity.

## 2. Materials and Methods

### 2.1. Patients and Samples

A total of 42 patients diagnosed with CLL (*n* = 39; 92.9%) and monoclonal B-cell lymphocytosis (*n* = 3; 7.1%) with available CBA, FISH and CMA results were included. Due to the purpose of the study, 18 of them presented CK by CBA (42.9%). All analyses were performed on cryopreserved peripheral blood mononuclear cells (PBMCs) obtained simultaneously to CBA or within one year. The median time from diagnosis to sampling was 10 months (range: 0–174 months), and only two patients (4.8%) received treatment prior to the genetic analyses. The main characteristics of the entire cohort are summarized in Table 1. The study was carried out in accordance with national and international guidelines (Professional Code of Conduct, Declaration of Helsinki) and approved by the Hospital del Mar Ethics Committee (2017/7565/I). Of note, CBA, CMA, and FISH data from 17 patients were included in a previous publication [28].

### 2.2. DNA Extraction, Labelling and Data Collection for Optical Mapping

For each sample, 1.5 million cells were used to extract ultra-high molecular weight (UHMW) DNA following manufacturer’s instructions (Bionano Prep Frozen Cells DNA Isolation Protocol, Bionano Genomics, San Diego, CA, USA). The Direct Label Enzyme (DLE-1) was used to enzymatically transfer DL-green fluorescent labeling to UHMW DNA following the Bionano Prep Direct Label and Stain (DLS) Protocol (Bionano Genomics). Labeled molecules were directly loaded onto a Saphyr chip and imaged via the Saphyr instrument (Bionano Genomics). The Saphyr Chip’s nano-channels allow only a single linearized DNA molecule to travel through via electrophoretic migration. Multiple cycles were run to generate approximately 1300 Gb of data per sample and reach an average genome coverage of 300×.

### 2.3. Data Assembly, Structural Variant Calling and Filtering

Genomic analysis was performed with the rare variant pipeline included in Bionano Solve software (v.3.5) and visualized with Bionano Access software (v1.6) (Bionano Genomics). Briefly, the imaged molecules were aligned against a reference assembly (hg19), and two algorithms were applied to identify SVs and large CNVs. The structural variant calling algorithm assessed any deviation in the labeling pattern to detect insertions, inversions, deletions, duplications, and intrachromosomal or interchromosomal translocations. Of note, interstitial deletions or inversions >5 Mb were called as intrachromosomal translocations by this pipeline. Copy number variant calls were inferred from the label coverage detected in each genomic interval, indicating the presence of gains or losses in those regions with a significant increase or decrease from baseline. All abnormalities called by the software were prefiltered according to default recommended confidence scores for Bionano Access version 1.6 (confidence scores: insertion: 0, deletion: 0, inversion: 0.7, duplication: −1, intra-translocation: 0.3, inter-translocation: 0.65, and CNV: 0.99) or whether they were described in the OGM control sample dataset provided by Bionano Genomics. In addition, as certain regions of the genome had an unusually high variance in their coverage that could be due to an unreliable molecule alignment (often concentrated around centromere and telomere regions), both pipelines masked SV and CNV calls occurring in these regions. Finally, we also applied size filters to further reduce the number of variants and obtain results comparable to those observed by CMA. The cut-off size for SVs (inversions, insertions, deletions, and duplications) was set at 100 Kb, while only those CNVs larger than 500 Kb were called by the CNV tool. Prefiltered SV and CNV data were downloaded and visually reviewed with the Bionano Access software in order to merge segmented CNVs, discard those SVs duplicated in the results, and variants found as benign polymorphisms in the Database of Genomic Variants (http://dgv.tcag.ca/dgv/app/home; accessed on 4 April 2022). In addition, the curated outputs from both algorithms were also compared in order to identify duplicities. In this regard, CNV gains and losses which overlapped with duplications and deletions or intrachromosomal translocation breakpoints, respectively, were considered as the same abnormality, and coordinates from the SV pipeline were taken. The final aberrations found in each patient were recorded for comparison with the results obtained by standard techniques. Additionally, less restrictive filters for confidence scores were applied in four patients with undetected known abnormalities to ascertain whether these were missed due to filtering issues or due to real OGM technical limitations.

### 2.4. Chromosome Banding Analyses and Fluorescence In Situ Hybridization

Peripheral blood (PB) samples were used to set two parallel cultures using phorbol-12-myristate-13-acetate (TPA) and immunostimulatory cytosine guanine dinucleotide (CpG)-oligonucleotide DSP30 plus interleukin 2 (IL-2) as mitogens. At least 20 metaphases from each culture were analyzed when possible, and a minimum of 10 were evaluated in three patients with abnormal karyotypes. Karyotypes from the most altered culture (more abnormal clones or altered metaphases detected) were collected and described according to the International System for Human Cytogenetic Nomenclature (ISCN 2020) [38]. The number and type of abnormalities were recorded as previously described [28].

Data from the routine FISH panel for *TP53* (17p13), *ATM* (11q22), D13S319 (13q14), and chromosome 12 centromeric region (Metasystems, Altlussheim, Germany) assessed in uncultured PB were also collected. In order to validate some of the OGM findings, additional FISH analyses were performed using commercial locus-specific, whole chromosome painting probes (Metasystems; Abbot Molecular, Abbott Park, IL, USA) or custom bacterial artificial chromosome (BAC) probes from the Children’s Hospital Oakland Research Institute library (Oakland, CA, USA). Details regarding the additional FISH probes tested are shown in Table S1.

### 2.5. Chromosomal Microarray Analyses

Chromosomal microarrays were assessed in DNA from PBMCs (*n* = 12) or PB CD19+ purified cells (*n* = 28). DNA was amplified, labeled, and hybridized to CytoScan HD (*n* = 30) or CytoScan 750K (*n* = 10) platforms (ThermoFisher Scientific, Eugene, OR, USA) according to manufacturer’s protocols. Copy number variants found as benign polymorphisms in the Database of Genomic Variants (http://dgv.tcag.ca/dgv/app/home; accessed on 4 April 2022) were excluded. The remaining aberrations, irrespective of size, were collected and reported using annotations of genome version GRCh37/hg19. CNV counting for risk classification of patients was performed using previously described criteria [27,28]. As the current analysis pipelines used for OGM results do not enable copy-number neutral loss of heterozygosity (CN-LOH) detection, these abnormalities were not considered in the present study.

### 2.6. Comparison among Techniques

First, we assessed the capability of OGM to detect the abnormalities previously defined by CBA, FISH, and CMA. In this regard, results obtained from standard techniques were compiled to set the list of abnormalities to be identified by OGM. The following criteria were applied: (i) for CNVs, coordinates defined by CMA were prioritized while FISH abnormalities or well-defined gains or losses identified in the karyotype but missed by CMA for technical reasons were also included; (ii) for balanced rearrangements (inversions or translocations), breakpoints defined in the karyotype were collected; (iii) among complex abnormalities in the karyotype affecting material of unknown origin, only the chromosomal breakpoints where additional material was identified were recorded to assess the presence of translocations by OGM in these regions. OGM results were considered concordant if the same abnormality was identified, even if it showed some variability in size or breakpoints. The percentage of detection by OGM was recorded, and potential causes of discrepancy were evaluated. Regarding additional abnormalities called by OGM, images from the available karyotypes and CMA data were reviewed to ascertain the potential relevance of OGM findings. Those abnormalities that could be compatible with any element observed in the karyotype were considered ‘real’ if validated by additional FISH analyses that allowed karyotype reinterpretation. In addition, rearrangements associated with chromothripsis events or CNVs detected by CMA were considered ‘real’ if validated by FISH or ‘potentially real’ if they were not specifically studied by FISH. Results from the additional FISH validations performed were used to finally classify some uncertain variants as ‘real’ or ‘false positive’ calls and to define custom criteria to identify novel OGM translocations with poor quality. The latter were classified as ‘potential false positive’ and were not considered in the final OGM abnormalities count.

In addition, we assessed the potential use of OGM for the identification of higher-risk patients based on their genomic complexity. To this end, the number of abnormalities reported by OGM was compared with that identified by standard methods in the whole cohort. Furthermore, patients were categorized based on the complexity detected by OGM and compared with the previously defined risk groups in the two ERIC studies [9,27].

### 2.7. Statistical Analyses

Descriptive statistics were used to provide frequency distributions of discrete variables, while statistical measures were used to provide median values and ranges for quantitative variables. Chi-square or Fisher exact tests were used to compare discrete variables and the Mann–Whitney U test to assess continuous variables. The number of abnormalities recorded by OGM and CBA or CMA were correlated by Spearman’s Rho, and the concordance among techniques was established via Cohen’s Kappa index. Time to first treatment (TTFT) was defined as the time interval between the sampling date and the date of first treatment. The Kaplan–Meier method was used to estimate the distribution of TTFT in the 40 treatment-naïve patients. Comparisons among patient subgroups were performed via the Log-rank test. The concordance statistic (C-index) for right-censored data was calculated to assess the accuracy of each technique in predicting TTFT. As only four patients died during follow-up, overall survival analyses were not assessed. Likewise, multivariate analysis could not be performed due to the limited size of the present cohort. Statistical analyses were performed using SPSS v.23 software (SPSS Inc, Chicago, IL, USA) and R v3.5.2 (R Foundation for Statistical Computing, Vienna, Arustria). *p*-values < 0.05 were considered statistically significant.

## 3. Results

### 3.1. Global SV and CNV Detection by OGM

OGM results from all 42 samples run met the recommended quality metrics, showing an average molecule N50 (for molecules > 150 Kb) value of 305 Kb and an average 363-fold effective coverage (Table S2). Globally, a total of 2202 abnormalities were called with the recommended prefiltering criteria for the Bionano Access version 1.6. Nonetheless, when the output was further filtered based on size (≥100 Kb) and visually reviewed, the number of detected abnormalities reduced to 579. In all, 36 deletions, 3 inversions, 4 duplications, 142 intrachromosomal translocations, 185 interchromosomal translocations, 59 CNV gains, and 150 CNV losses were considered for comparison with standard techniques. Of note, 42 of these CNVs were also called as duplications (*n* = 6), deletions (*n* = 24), or intrachromosomal translocations (*n* = 12) by the SV calling pipeline and subsequently not counted in the genomic complexity estimation (Figure S1).

### 3.2. Concordance Rate of OGM Results with Standard Techniques

In the whole cohort, a total of 309 abnormalities previously detected by CBA, FISH, and/or CMA were considered to explore the concordance with OGM results. While CNVs represented the vast majority of these (193 losses and 67 gains), information from only 49 translocations was previously available. Of note, in 29 of the rearrangements, only one of the translocation breakpoints was identified by CBA, as they involved additional material of unknown origin in the karyotype (“add”). Globally, 90.3% of the previously known alterations (279/309) could be identified by OGM. Of note, no significant differences in the detection rate were observed between patients with non-CK and those carrying a CK by CBA (detection of 83/89 (93.3%) and 196/229 (89.1%), respectively; *p* = 0.396). In order to reach this concordance, a lower confidence score threshold was applied to properly detect five known translocations, and more permissive filtering criteria were used to pick up 15 alterations initially missed by the CNV pipeline. The latter included nine small CNVs (range: 292–488 Kb) filtered by the 500 Kb cut-off size used for CNVs, and six larger CNVs (range: 1282–22,049 Kb), which had been detected in less than 20% of cells by FISH and/or CMA and could also be visually suspected in the whole chromosome CNV view of the Bionano Access software. It is noteworthy that, when comparing only those abnormalities included in the routine CLL FISH panel, the concordance rate increased to 95.6% (detection of 55/57 known deletions on 13q14, 11q22 (*ATM*), 17p13 (*TP53*) or trisomy 12). In this regard, OGM failed to detect a 13q14 deletion and a *TP53* deletion found in 15% and 17% of nuclei by FISH, respectively, and did not pick up any additional one of these recurrent CLL alterations.

As well as their detection, we could evaluate OGM’s accuracy regarding the definition of genomic location and size for 238/247 CNVs previously defined by CMA. To this end, CNVs detected by both techniques showed a high overlap with a median difference of only 22 Kb in the defined coordinates (range: 0–8.1 Mb). Major discordances were due to the known OGM masking of the terminal 2–5 Mb from large CNVs or whole chromosome aneuploidies, which were located at repetitive regions (12/238, 5.0%). Once these discrepancies found to be masked were excluded, differences larger than 2 Mb in size (range: 2.1–5.7 Mb) were only observed in five CNVs (2.1%). It is noteworthy that OGM allowed the definition of the genomic coordinates from eight known CNVs that had been previously detected by FISH and/or CBA but missed by CMA. In addition, similar results were observed when the alterations called by the SV and CNV pipelines were compared.

Among the 30 missed abnormalities, 14 were not expected to be detected by OGM as they were translocations with breaks in the (peri-)centromeric or telomeric regions (*n* = 11), which are not covered by OGM labels, two relatively small telomeric deletions (1.5 and 2.5 Mb) and one gain involving chromosome Y (4.1 Mb) that were masked by the CNV analysis pipeline to avoid the calling of false positive in these highly variable regions. On the other hand, sensitivity explained the discrepancy in 13 of the remaining 16 aberrations that were susceptible to detection by OGM (Table 2). In this regard, nine CNVs undetected by OGM were identified in a median of 17% of cells by FISH and/or CMA (range: 3–25%). Among these, three were not detected by CMA either. Similarly, sensitivity could explain four missed translocations that had been detected in minor clones by CBA related to the culture selection of abnormal clones intrinsic to this technique. Although the percentage of abnormal cells could not be set for these translocations, it is important to note that the SV pipeline correctly detected some other abnormalities found in only 5% of cells by FISH. Finally, OGM was not able to identify a small 138 Kb deletion encompassed in a complex chromothripsis event and two additional abnormalities that could not be associated with any feasible cause of discrepancy (Table 2).

Detailed results obtained by each of the standard techniques, the 309 known abnormalities considered, and their detection by each of the OGM analysis pipelines are shown in Table S3.

### 3.3. Novel Abnormalities Detected by OGM

**Figure 1:**
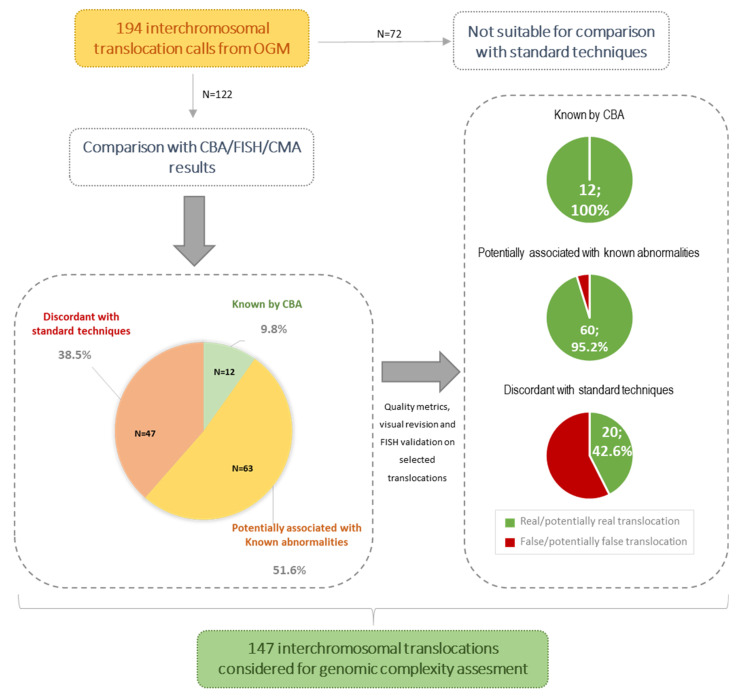
Summary of the analyses performed to define the final set of interchromosomal translocations considered as real or potentially real in the present cohort.

**Figure 2:**
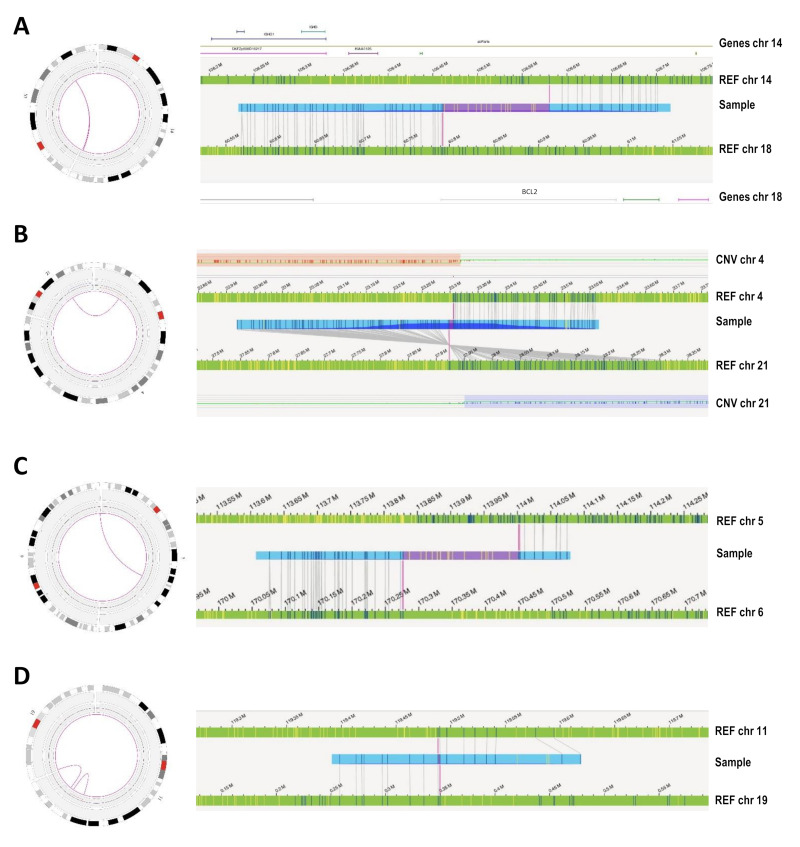
Examples of the Circos plots and genome map views showing two real and two non-validated interchromosomal translocations. Validated translocations displayed a high number of labels mapped on each chromosome involved in the rearrangement: (**A**) Previously known t(14;18)(q32;q21) from patient #16, in which OGM also correctly identified the involvement of the *BCL2* and IGH genes in the rearrangement, and (**B**) t(4;21) with breakpoints at the limit of known CNV by CMA detected in patient #12, which corresponded to an unbalanced translocation that was cryptic in the karyotype and could be validated by WCP. On the contrary, images (**C**,**D**), representing a t(5;6) found in patient #3 and a t(11;19) from patient #13, were not validated by FISH using custom BAC probes for the specific rearrangements. These calls showed a low number of labels (<10) in at least one of the mapped chromosomes, which corresponded to the one showing low confidence score values. Of note, none showed breakpoints affecting regions provided by Bionano Genomics as DLE-1 masked (such as segmental duplications, common false positive regions or gaps).

As OGM provided data not only on CNV genomic location but also structural information, we next assessed whether OGM could provide a more comprehensive structural overview of the known aberrations detected compared to a CBA, FISH, and CMA combination. Of note, the number of interchromosomal translocations called by OGM (*n* = 194) was much higher than expected, taking into account previous CBA data, even in non-CK patients. In this regard, additional analyses were performed to ascertain which calls should be considered real or, on the contrary, filtered as potential false-positive calls. A total of 72 translocations were considered unsuitable for these focused comparisons as they were distributed among only three patients (with 16, 20, and 36 translocations, respectively) that showed highly complex karyotypes including several abnormalities involving material of unknown origin, difficult to compare with OGM or validated by FISH. Thus, these three cases were excluded from the comparison. Among the remaining 122 translocations with CBA and CMA data available, 12 (9.8%) corresponded to previously well-defined translocations (seven known translocations, five of them having two breakpoints identified in each derivative chromosome), and 63 (51.7%) could potentially be reflecting unknown rearrangements associated with previously described abnormalities. Conversely, 47/122 (38.5%) were not supported by CBA and were called in apparently normal regions by CMA (Figure 1). Visual inspection of OGM calls revealed that, contrarily to that observed for those concordant or potentially real calls, most apparently false-positive calls displayed few labels mapped in at least one of the translocation partners (≤10), which also correlated with low ‘RawConfidenceLeft’ or ‘RawConfidenceRight’ parameter values (<10) in the SMAP file generated by the rare variant pipeline (Figure 2). Three of these translocations with low labels [t(5;6)(q22.3;q27) called in patients 3 and 5, and t(11;19)(q23.3;p13.3) in patient 13] were assessed by interphase FISH using targeted custom BACs break-apart probes and could not be validated. Moreover, 51 additional calls with high label coverage in both translocation partners were indirectly validated by whole chromosome FISH painting (Table S4). These findings suggested that the final set of translocation calls to be considered could be manually filtered based on these criteria. On the other hand, high variability was observed in the number of self-molecules supporting the translocations called by OGM (median: 45; range: 5–175). A total of 27 of the apparently well-covered translocations were based on less than 10 molecules, which is the cut-off used by some authors in hematological studies [39,40]. Thereby, we next evaluated if these low values could also be associated with a higher rate of false-positive calls, but no conclusive results were obtained. Fourteen translocations were consistent with CBA or whole chromosome FISH painting (WCP) information, whereas two were not validated by interphase FISH with custom BAC probes and were classified as false-positive calls. Notably, five of the latter were subclonal abnormalities by CBA detected in only 5–7% of nuclei by FISH or confirmed in clones initially missed by CBA (Table S4). These results suggested that the number of molecules supporting the translocation calls could be impaired by both potential errors in the mapping of the sample or by intrinsic characteristics of the abnormal clone. Thus, this parameter was not considered in the final filtering of the translocations found in the whole cohort. Likewise, contrarily to that described by other authors [37], as most of the validated translocations failed the assembly chimeric score provided by the software, this parameter was not considered either. Globally, 45/192 (23.4%) interchromosomal and 5/142 (3.5%) intrachromosomal translocation calls were manually discarded from the analysis, and none corresponded to any known abnormality by conventional techniques (Figure 1).

**Figure 3:**
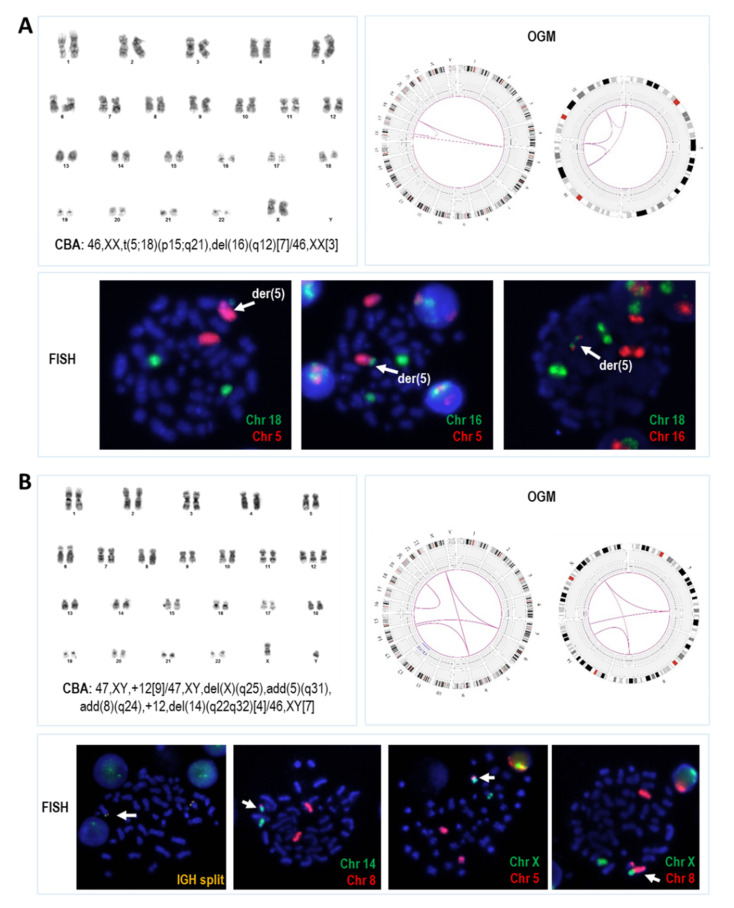
Examples of cases in which OGM revealed that abnormalities apparently well-described by CBA were more complex than initially assumed. (**A**) Patient #24 showed an abnormal karyotype described as containing a t(5;18)(p15;q21) and a del(16)(q12). Whole-genome Circos plot and Circos plot from chromosomes 5, 16 and 18 obtained by OGM revealed that the initially reported abnormalities were a three-way translocation between these chromosomes. OGM results were confirmed by WCP, in which material from both chromosomes 16 and 18 was detected in the der(5) chromosome; (**B**) The karyotype defined in patient #CK11 contained two deletions [del(X)(q25) and del(14)(q22q32)] and additional material of unknown origin at chromosomes 5 and 8 [add(5)(q31) and add(8)(q24)]. Circos plots show the four translocations identified by OGM that shared breakpoints on chromosomes X, 5 and 8, and two breakpoints on chromosome 14 were located at the start and end coordinates from a known CNV loss identified by CMA. FISH analysis allowed their validation and karyotype reinterpretation. The corrected ISCN formula for this abnormal clone was finally defined as 47,XY,der(X)t(X;5)(q22.1;q32),der(5)t(5;14)(q32;q32.2),der(8)t(X;8)(q22.1;q23.3),+12,der(14)t(8;14)(q23.3;q24.1).

Once data were further manually filtered, novel structural findings on previously known aberrations were found in 23/42 patients (54.8%). On the one hand, when compared with CBA, OGM identified 23 intra- and 37 interchromosomal translocations associated with 33 of the previously known structural rearrangements: 15 called as translocations, inversions, insertions, or deletion, and 18 reported as “add” in the karyotype. Interestingly, OGM showed rearrangements involving at least three chromosomes in 13/18 (72.2%) abnormalities in which only one of the breakpoints could be identified in the karyotype (“add”). Moreover, it also revealed higher complexity in 7/15 (46.7%) apparently well-described abnormalities (Figure 3). On the other hand, 93 intra- and 30 interchromosomal translocations underscored by OGM were associated with CNVs identified by CMA. Apart from unbalanced rearrangements underlying large terminal CNV or rearrangements associated with complex chromothriptic events, unexpected rearrangements between apparently unrelated small interstitial CNV were also recorded. Interestingly, apart from the three translocations with breakpoints at 13q14 defined by CBA, OGM detected novel translocations associated with known 13q14 or 11q22 (including *ATM*) deletions in 5/29 (17.2%) and 2/11 (18.2%), respectively (Figure S2).

In addition to the aforementioned translocation calls, OGM also identified three inversions and six deletions ≥100 Kb previously undetected by conventional techniques. Among these deletions, three could be identified in the revision of raw CMA data (sizes: 0.8, 1.8, and 4.1 Mb), and one was validated by FISH (6% of the nuclei presented the deletion using a custom BAC probe within the 4.2 Mb region lost by OGM). The two remaining discordant deletions were of just 230 Kb and 239 Kb and were not further assessed. In addition, in the two patients with no CMA study available, OGM underscored the presence of two large CNVs associated with a cryptic translocation by CBA (validated by FISH) and a 1.2 Mb deletion associated with a known t(13;17)(q14;p13).

### 3.4. Global Genomic Complexity Found by OGM

Regarding the genomic complexity identified by OGM, after the filtering and manual revision of raw results, the median number of abnormalities among the whole cohort was five (range: 1–70). As expected, those patients showing a CK by CBA displayed a significantly higher median number of alterations compared with non-CK patients (20 [range: 4–70] vs. 3 [range: 1–48]; *p* < 0.001). These differences were related to higher detection of both translocations and CNVs in the CK group (Figure S3). A significant strong correlation with the number of anomalies recorded by CBA and CMA was observed (r_s_ = 0.734 and r_s_ = 0.845, respectively). Nonetheless, it is noteworthy that OGM detected a significantly higher complexity than conventional techniques, which detected a median of two and three abnormalities by CBA and CMA, respectively (range: 0–16 for CBA and 0–30 for CMA) (Figure S3). In terms of prognostic impact, considering the number of abnormalities as a continuous variable, OGM presented a higher accuracy in predicting TTFT than CBA and CMA (C-index: 0.696, 0.602, 0.661; by OGM, CBA, and CMA, respectively).

**Figure 4:**
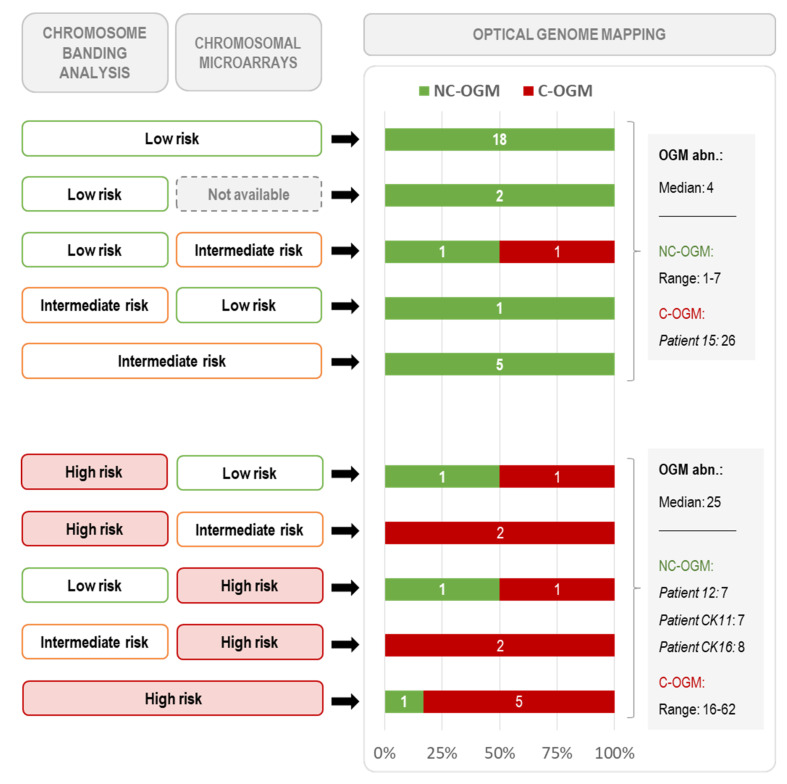
Patient distribution based on genomic complexity risk stratification by CBA, CMA and OGM. CBA and CMA categories were defined as described in previous studies (Ramos-Campoy et al., 2022): Low risk (0–2 abnormalities), Intermediate risk (3–4 abnormalities) and High risk (≥5 abnormalities). Patients were divided into two categories based on OGM results: NC-OGM (Non-complex by OGM, <10 abnormalities) and C-OGM (Complex by OGM, ≥10 abnormalities). Each line represents one possible combination of the risk assigned by CBA and/or CMA (depicted on the left), for the OGM results (right) the number of patients classified into each category is plotted and the number of abnormalities recorded is detailed in the gray boxes. Globally, for the identification of high-risk patients, a moderate-strong agreement was observed between OGM and the combination of CBA and CMA methods (κ = 0.778; *p* < 0.001).

Next, we evaluated if OGM could be a useful tool for risk stratification based on genomic complexity in CLL. Thus, considering the abnormalities found by OGM and comparing their distribution among the CBA and CMA risk categories defined in previous studies [28], an arbitrary cut-off of ≥10 alterations was used to define two risk groups by OGM. Based on this criterion, genomic complexity by OGM was assigned to 12 patients (28.6%) (Complex by OGM, C-OGM), while the remaining 30 patients were classified into a lower risk category (Non-complex by OGM, NC-OGM). Globally, the C-OGM group included 11/14 patients considered to be in the highest risk category by at least one standard technique (CBA and/or CMA) and one additional patient with a normal karyotype that, despite being considered to be in the CMA intermediate-risk group, displayed chromothripsis (Figure 4). Detailed OGM information from the three higher-risk patients, according to conventional methods, not included in the C-OGM group is shown in Figure 3B (patient CK11) and Figure S4 (patients 12 and CK16). Globally, for the identification of higher-risk patients, a moderate-strong agreement was observed between OGM and the combination of CBA and CMA methods (κ = 0.778; *p* < 0.001). As expected, the C-OGM group also showed an increased proportion of patients with *TP53* abnormalities (58.3% vs. 3.3%, *p* < 0.001) and a significantly shorter TTFT (median TTFT: 2 vs. 43 months, *p* = 0.014) compared with NC-OGM patients (Figure S5). Conversely, none of the other patient characteristics compared presented significant differences (Table 3). Interestingly, all patients with multiple trisomies by CBA (namely +12, +19, +/− other abnormalities; patients 21, CK1, CK15, CK18), previously described to exhibit very favorable outcomes [24], were categorized into the NC-OGM group with four to eight abnormalities detected by this technique.

## 4. Discussion

Over the last decade, several works have highlighted the prognostic and predictive value of genomic complexity in patients with CLL. Recent large ERIC cooperative studies have validated the performance of both CBA and CMA in the risk stratification of CLL and suggested the detection of five or more chromosomal abnormalities as an adverse biomarker to be implemented in routine CLL management [9,27]. As both techniques had some intrinsic limitations that led to discordant risk stratification in up to 30% of patients [28], herein, we aimed to evaluate whether the recently developed OGM technology is a suitable alternative to overcome these discrepancies. To the best of our knowledge, this is the largest cytogenetic characterization via OGM conducted to date in CLL patients.

Overall, we demonstrated the feasibility of OGM in detecting 90% of the alterations previously defined by the combination of standard techniques (CBA, FISH, and/or CMA) in a single assay. In addition, this technique afforded not only high accuracy in the definition of genomic coordinates for CNVs when compared with CMA but also provided additional structural information on previously known aberrations. As expected, the concordance reached was similar to that reported in other hematological malignancies, in which OGM detection of clinically relevant known abnormalities ranged between 90 and 100% [35,36,37,40,41,42]. The number of missed abnormalities in the present series was low, but the discrepancies found underscore some OGM limitations that should be taken into account. First, although this is a whole-genome view technology, the analysis is compromised at some locations that could not be properly labeled or mapped (such as centromeres, telomeres, or regions with a high number of repetitions). Thus, nearly half of the 30 abnormalities missed in our study involved these regions and were not expected to be detected by OGM. Considered individually, as none had been previously described as clinically relevant in CLL, their misdetection is not assumed to be highly relevant for patient management. However, as suggested by other authors, manual inspection of OGM imaged molecules is highly recommended for pathologies in which aberrations affecting these regions are well-known prognostic biomarkers (e.g., *CRLF2* rearrangements involving the PAR1 region on chromosome X in acute lymphoblastic leukemia) [37,41,42]. It is noteworthy that these studies were performed against hg19 [GRCh37], as used herein, and some cases would be identified using hg38 [GRCh38] as a reference, which has a more robust assembly for the Xp region [43]. Second, OGM specifications describe that at 300x effective coverage, it allows the detection of SVs and CNVs in up to 5% and 10% of the allele fraction, respectively. In our experience, the SV tool was able to detect some translocations at these low frequencies, but the detection of CNVs was not fully guaranteed if present in less than 20% of cells. In this regard, sensitivity was assumed to be the most feasible cause of the discrepancy in 43% of the missed abnormalities. While these variants had been previously detected in a median of 17% of cells, concordant OGM results also included CNVs found in only 12–15% of nuclei by FISH and/or CMA. Although some authors have set the sensitivity threshold for OGM at 10% [35,41], our results were in accordance with Rack et al. and suggested that detection of abnormalities around or below 15% varies among regions or loci [37]. It is noteworthy that some of the CNVs not called by OGM were visually identified in the whole-genome CNV view with Bionano Access and could potentially be manually added in future software upgrades, as currently performed for CMA analysis.

In contrast to other published OGM studies in hematological malignancies, which focused on the detection of clinically relevant abnormalities, our goal was to evaluate CLL abnormalities genome-wide in order to identify patients with genomic complexity. This strategy allowed us to benefit from the high potential of OGM, underscoring novel aberrations which had not been identified with standard methods and whose clinical relevance deserves further analysis. On the one hand, OGM identified several small SVs (from 3 Kb to 100 Kb) that were considered beyond the scope of this study. Our proof-of-concept analysis aimed to obtain comparable results with the currently used methodologies; therefore, a cut-off size of 100 Kb was set to generate results with a resolution similar to that of CMA. Nonetheless, these small aberrations missed by standard methods could involve clinically relevant genes (novel or already known) and should be analyzed in more depth in subsequent studies and in larger CLL cohorts. On the other hand, a higher number of chromosomal translocations than initially expected was detected by OGM. Indeed, novel translocations allowed us to better characterize known abnormalities in 54.8% of the patients assessed. These included not only abnormalities involving material of unknown origin by CBA (marker chromosomes or “add”) but also provided novel structural information related to apparently well-characterized translocations or deletions in the karyotype, as well as CNVs detected by CMA. Notably, not all the novel OGM translocations correlated with the results defined by standard techniques, and nearly 20% of them were classified as false-positive findings. Other authors have also reported similar observations and suggested manual inspection to filter out SV artifacts distinguishable from true translocation calls [35,36,37]. Following our experience, those translocation calls based on the mapping of ≤10 labels in one of the translocation partners should be filtered. In contrast, while we were not able to validate two translocations with less than 10 molecules, 22% of the previously known or validated translocations were supported by only five to nine mapped molecules by OGM. Thus, contrarily to some authors that set a minimum of 10 supporting molecules for the analyses [39,40], we recommend not filtering these variants but performing validations with alternative techniques before reporting them. It is remarkable that, since we processed the samples, a new upgrade of Bionano Access has been developed that has improved the filtering of these types of false-positive calls. In this regard, in two of our samples reprocessed with the most recent update of Bionano Access (1.7 version), nine of the ten inter-translocations assumed as potential false-positive results were no longer called, but novel translocations were also identified in this updated version (data not shown). Globally, we have demonstrated that the custom filtering criteria applied herein are useful for OGM analysis, but the distinction between true translocations and technical artifacts is sometimes challenging. Consequently, guidelines for the filtering and interpretation of OGM variants are urgently needed to implement this technique in a routine setting.

Concerning genomic complexity assessment by OGM, as expected by its high resolution, our results demonstrated that this novel technique was able to identify an overall higher number of abnormalities than conventional methods. Nonetheless, when data were manually curated, and small SVs (<100 Kb) were excluded from the analysis, a strong correlation between techniques was found. Likewise, we demonstrated that occasional discrepancies in the detection of known aberrations did not hinder the accuracy of the number of abnormalities recorded by OGM in predicting TTFT, which was even better than that achieved by CBA and CMA. Indeed, the higher C-index obtained by OGM indirectly proves the clinical importance of the additional aberrations identified by OGM, which allows a better delineation of the genomic complexity of these patients compared with current methodologies. Apart from its predictive value as a continuous variable, the definition of standard criteria to discriminate those higher-risk patients based on complexity is mandatory to incorporate OGM technology in the standard diagnostic algorithm. In this regard, we were able to demonstrate that 91.7% of patients harboring at least 10 abnormalities by OGM (C-OGM group) were also classified into the highest risk group based on the complexity identified by the combination of CBA and CMA. Moreover, this C-OGM group showed a statistically significantly higher proportion of *TP53^del/mut^* cases and shorter TTFT. It is noteworthy that, as the cohort analyzed herein is limited in size and highly enriched in patients carrying CK, the cut-off used could not be statistically set. Thus, it should only be taken into account as a proof-of-concept of the capability of OGM to stratify patients based on their genomic complexity. Further studies in larger cohorts of CLL patients are required to statistically define robust criteria for genomic complexity stratification by OGM, not only in retrospective series but also to clarify its potential role in the management of patients treated with novel targeted agents.

On the other hand, OGM represents a cost-effective approach to substitute CBA combined with multiple FISH tests that reduce global costs and turn-around times in other hematological malignances (e.g., acute lymphoblastic leukemia and acute myeloid leukemia). In contrast, the total expenses of the OGM technique (technician time dedication, reagents, equipment, and other technical infrastructure required for its implementation) are still superior to those of CBA; currently, the standard-of-care method to detect genomic complexity CLL. Thus, major benefits from OGM application in CLL do not rely on the cost savings but on the better genomic characterization achieved and novel abnormalities that could be uncovered from these highly complex genomes.

## 5. Conclusions

In summary, we have confirmed that OGM is a valuable tool for the cytogenomic assessment of CLL patients. It not only effectively detects most of the abnormalities defined by the combination of standard methods in a single test but also provides a more comprehensive genomic analysis allowing an enhanced interpretation. Despite a few abnormalities still being missed due to OGM limitations, these are expected to be addressed by the technical and analytical improvements of upcoming Bionano Access upgrades. As for its utility for risk stratification, herein, for the first time, we have demonstrated the association of an increasing number of abnormalities by OGM with a worse clinical evolution of CLL patients. Nonetheless, further studies in larger unselected cohorts are required to define standard genomic complexity criteria by OGM and to define the clinical significance of the novel abnormalities underscored by this technology. In conclusion, OGM is a new methodology that could potentially replace current cytogenomic methodologies for the routine management of CLL patients in the future.

## Figures and Tables

**Table 1:** Baseline characteristics of patients at diagnosis and last follow-up.

	Non-CK Group	CK Group	*p*-Value
	*n* = 24; *n* (%)	*n* = 18; *n* (%)	
**Gender**			
Male	17 (70.8%)	11 (61.1%)	0.530
**Median age at diagnosis (range)**	66 (42–85)	69 (37–88)	0.297
**Stage at diagnosis**			
MBL	0 (0.0%)	3 (16.7%)	0.071
CLL	24 (100%)	15 (83.3%)	
Binet A	22 (91.7%)	10 (66.7%)	0.085
Binet B/C	2 (8.4%)	5 (33.3%)	
Common CLL genomic aberrations *			
del(13)(q14)	19 (79.2%)	11 (61.1%)	0.302
Trisomy 12	3 (12.5%)	5 (27.8%)	0.256
del(11)(q22q23)	5 (20.8%)	7 (38.9%)	0.302
Aberrations in *TP53*	0 (0.0%)	8 (44.4%)	<0.001
del(17)(p13)	0 (0.0%)	7 (38.9%)	0.001
*TP53* mutation	0 (0.0%)	7 (38.9%)	0.001
**Unmutated IGHV**	12/23 (52.2%)	12/18 (66.7%)	0.524
**Time from diagnosis to cytogenetic study (range)**	22.5 months (0–123)	6 months (0–174)	0.867
**Median follow-up (range)**	44.5 months (0–95)	31.5 months (0–78)	0.065
**Treatment**			
Treated patients	13 (54.2%)	13 (72.2%)	0.338
Median time to first treatment (95% CI)	43 months (26–54)	9 months (8–30)	0.165

* Deletions and trisomy detected by FISH and/or chromosomal microarray analysis. Abbreviations: MBL = monoclonal B-cell lymphocytosis, CI = confidence interval.

**Table 2:** Known abnormalities not detected by OGM and their potential cause of discrepancy.

Patient ID	Undetected Alteration	Size (Kb)	Potential Cause of Discrepancy	Comments
12	Translocation t(13;?)(p11;?)	-	Involvement of (peri-)centromeric regions	
CK4	Translocation t(17;?)(p11;?)	-	Involvement of (peri-)centromeric regions	
CK8	Translocation t(17;18)(q10;q10)	-	Involvement of (peri-)centromeric regions	
CK9	Translocation t(13;21)(q11;p11)	-	Involvement of (peri-)centromeric regions	
CK10	Translocation t(14;?)(p11;?)	-	Involvement of (peri-)centromeric regions	
CK12	Translocation t(11;?)(p11;?)	-	Involvement of (peri-)centromeric regions	
CK13	Translocation t(14;17)(q11;p11)	-	Involvement of (peri-)centromeric regions	
CK16	Translocation t(14;18)(p11;q11)	-	Involvement of (peri-)centromeric regions	
CK16	Translocation t(15;22)(p11;q15)	-	Involvement of (peri-)centromeric regions	
CK17	Translocation t(15;?)(p11;?)	-	Involvement of (peri-)centromeric regions	
CK4	Translocation t(6;19)(q12;p13)	-	Involvement of telomeric region	
7	Gain Yq11.223q11.23(24637115_28799654)	4163	Masked region (partial CNV on chr. Y)	Detected at 50% by CMA, and visually suggested in the whole genome CNV view
CK2	Deletion 17p13.3p13.3(525_2489182)	2489	Masked region (telomere)	
CK6	Deletion 1p36.33p36.32(1997349_3740109)	1743	Masked region (telomere)	
20	Deletion 6q21	NA	Sensitivity	Detected in 12% of nuclei by FISH (CMA not available)
21	Deletion 13q14 (D13S319)	NA	Sensitivity	Detected in 17% of nuclei by FISH (not detected by CMA)
CK3	Gain 6pterq16	NA	Sensitivity	Detected in 3% of nuclei by FISH, confirmed in metaphases (not detected by CMA)
CK8	Deletion 17p13.3p11.2(526_21347924)	21347	Sensitivity	Detected in 15% of nuclei by FISH (also visually found by CMA)
CK12	Gain 11q14.2q14.2(86177075_86856206)	679	Sensitivity	Detected at 25% by CMA, and visually suggested in the whole genome CNV view
CK12	Gain 11q22.3q22.3(106600681_106991146)	390	Sensitivity	Detected at 25% by CMA, and visually suggested in the whole genome CNV view
CK12	Gain 11q24.3q24.3(129345165_130249509)	904	Sensitivity	Detected at 25% by CMA, and visually suggested in the whole genome CNV view
CK12	Gain 19q13.2q13.42(41644540_54499334)	12855	Sensitivity	Detected at 20% by CMA
CK13	Gain 1p22pter	NA	Sensitivity	Detected in 5% of nuclei by FISH (not detected by CMA)
7	Translocation t(X;?)(p22;?)		Sensitivity	Percentage not available, could be a minor clone expanded during CBA culture
CK4	Translocation t(12;?)(q24;?)		Sensitivity	Percentage not available, could be a minor clone (“add(12)(q24)” detected as clonal evolution in only two out of eight abnormal metaphases)
CK6	Translocation t(9;?)(q34;?)		Sensitivity	Percentage not available, could be a minor clone expanded during CBA culture (CK defined as a composite karyotype)
CK8	Translocation t(6;?)(p25;?)		Sensitivity	Percentage not available, could be a minor clone (“add(6)(p25)” detected as clonal evolution from abnormal cells with monosomy of chr. 17, as the *TP53* deletion was detected at 15% the abnormality was probably below that percentage)
8	Deletion 13q32.1(95520821_95658848)	138	Small deletion within chromothripsis	Small deletion, part of a complex CMA profile on chr. 13 properly detected
CK16	Translocation t(11;?)(q23;?)		Unknown cause of discrepancy	The “add(11)(q23)” was present in the main clone. Although not called as SV, some imaged molecules showed fusions between different regions of chr 11; WCP for chr. 11 confirmed hybridization in the whole abnormal chromosome
CK17	Translocation t(9;?)(q34;?)		Unknown cause of discrepancy	Percentage not available; although the “add(9)(q34)” could be a minor clone (detected as clonal evolution in only two out of 13 abnormal metaphases), other abnormalities from the same clone were properly detected. WCP revealed that the additional material was from chr. 17 and, as seen by CBA, the chr. 17 telomeric region could be involved in the fusion (telomeric regions are masked by the SV pipeline)

Abbreviations: NA = Not available; WCP: whole chromosome FISH painting.

**Table 3:** Comparison of the clinical and biological characteristics of CLL patients classified according to the genomic complexity detected by OGM.

Characteristics	NC-OGM (*n* = 30)	C-OGM (*n* = 12)	*p*-Value
**Age at diagnosis**	68 (37–85)	67 (55–88)	0.944
**Males**	20 (66.7%)	8 (66.7%)	1.000
**Advanced Binet stage (B or C)**	3 (10.0%)	4 (33.3%)	0.088
**Time from diagnosis to OGM study (months)**	8 (0–174)	25 (0–125)	0.854
**Number of abnormalities by OGM (filtered data)**	4 (1–11)	32 (16–70)	<0.001
Copy number variants (CNV gains and losses)	1 (0–4)	12 (2–25)	<0.001
Translocations (intra and interchromosomal)	2 (0–8)	22 (9–42)	<0.001
**Genomic complexity by conventional methods**			
Low/intermediate-CK by CBA (3–4 abn.)	6 (21.4%)	2 (7.1%)	1.000
High-CK by CBA (≥5 abn.)	2 (6.7%)	8 (66.7%)	<0.001
Intermediate-GC by CMA (3–4 abn. *) (*n* = 40)	6/28 (21.4%)	3/12 (25.0%)	1.000
High-GC by CMA (≥5 abn. *) (*n* = 40)	2/28 (7.1%)	8/12 (66.7%)	0.001
High complexity by CBA and/or CMA (≥5 abn.)	2 (6.7%)	11 (91.7%)	<0.001
Chromothripsis	0 (0.0%)	9 (75.0%)	<0.001
**FISH abnormalities**			
del(13q)	22 (73.3%)	8 (66.7%)	0.715
Trisomy 12	7 (23.3%)	1 (8.3%)	0.402
del(11q) [*ATM*]	8 (26.7%)	4 (33.3%)	0.715
del(17p) [*TP53*]	1 (3.3%)	6 (50.0%)	0.001
***TP53* abnormalities (del/mut)**	1 (3.3%)	7 (58.3%)	<0.001
**Unmutated IGHV (*n* = 41)**	15/29 (51.7%)	9/12 (75.0%)	0.296
**Last follow-up (*n* = 40) ^¥^**			
Treated patients	16/30 (53.3%)	8/10 (80.0%)	0.090
Time to first treatment (months, 95% CI)	43 (28.0–52.6)	2 (1.9–23.0)	0.014
Follow-up (months)	42 (0–95)	26 (0–82)	0.070

Values are given as median (range) or number (%). Abbreviations: NC-OGM = non-complex by optical genome mapping, <10 abnormalities), C-OGM = complex by optical genome mapping, ≥10 abnormalities), CBA = Chromosome banding analysis, CMA = Chromosomal microarray analysis, abn. = abnormalities. * Criteria defined by Leeksma et al., 2020 for intermediate genomic complexity (Intermediate-CG): 3–4 CNV; and high genomic complexity (High-CG): ≥5 CNV, including all classic CLL CNV (chromosome 12 and losses of 11q, 13q and 17p) irrespectively of their size and other CNVs > 5 Mb. ^¥^ Those patients treated prior to genetic testing were excluded from the time to first treatment analyses.

## Data Availability

Detailed chromosome banding analyses, chromosomal microarrays and optical genome mapping profiles are provided in Supplemental Tables. Additional data of the study are available from the corresponding authors on reasonable request.

## Supplementary Materials

The following supporting information can be downloaded at: https://www.mdpi.com/article/10.3390/cancers14143376/s1, Figure S1. Flow diagram for filtering SV and CNV calls to define the final set of OGM abnormalities to be compared with standard techniques. Figure S2. Examples of novel translocations called by OGM that were associated with known CNVs previously identified by CMA. Figure S3. Distribution of the number of abnormalities detected in non-CK and CK groups. Figure S4. Example of two of the high-risk patients by CBA and/or CMA with less than 10 abnormalities detected by OGM. Figure S5. Kaplan–Meier plots for time to first treatment (TTFT) based on the presence of genomic complexity detected by OGM. Table S1. FISH probes tested to validate OGM results. Table S2. Technical performance of OGM in the whole cohort. Table S3. Detailed information on the detection of the 309 known abnormalities by OGM. Table S4. Summary of the interchromosomal translocation calls detected by OGM and comparison with CBA and FISH data.
