# Supplementary material for: Optical Genome Mapping: A Promising New Tool to Assess Genomic Complexity in Chronic Lymphocytic Leukemia (CLL)
Source: Cancers (Basel). 2022 Jul 11;14(14):3376. doi: 10.3390/cancers14143376 (PMC9317182; doi:10.3390/cancers14143376)
Supplement: Supplementary file 1 [file cancers-14-03376-s001.zip › cancers-1793703-Supplementary Figures.pdf]

cancers

MDPI

Supplementary Materials

Optical Genome Mapping: A Promising New Tool to Assess Genomic Complexity in Chronic Lymphocytic Leukemia (CLL)

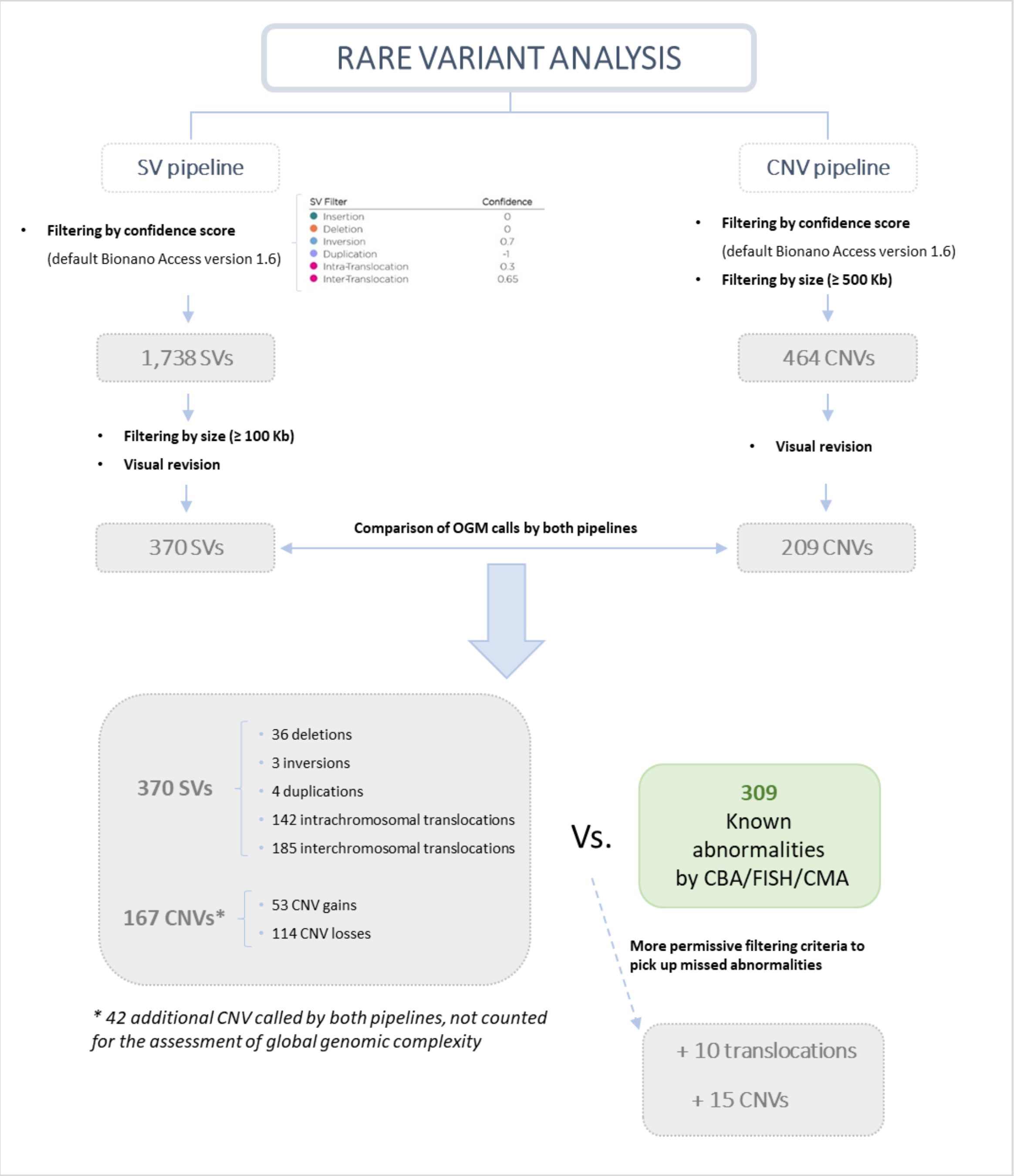

Figure S1. Flow diagram for filtering SVs and CNVs calls to define the final set of OGM abnormalities to be compared with standard techniques.

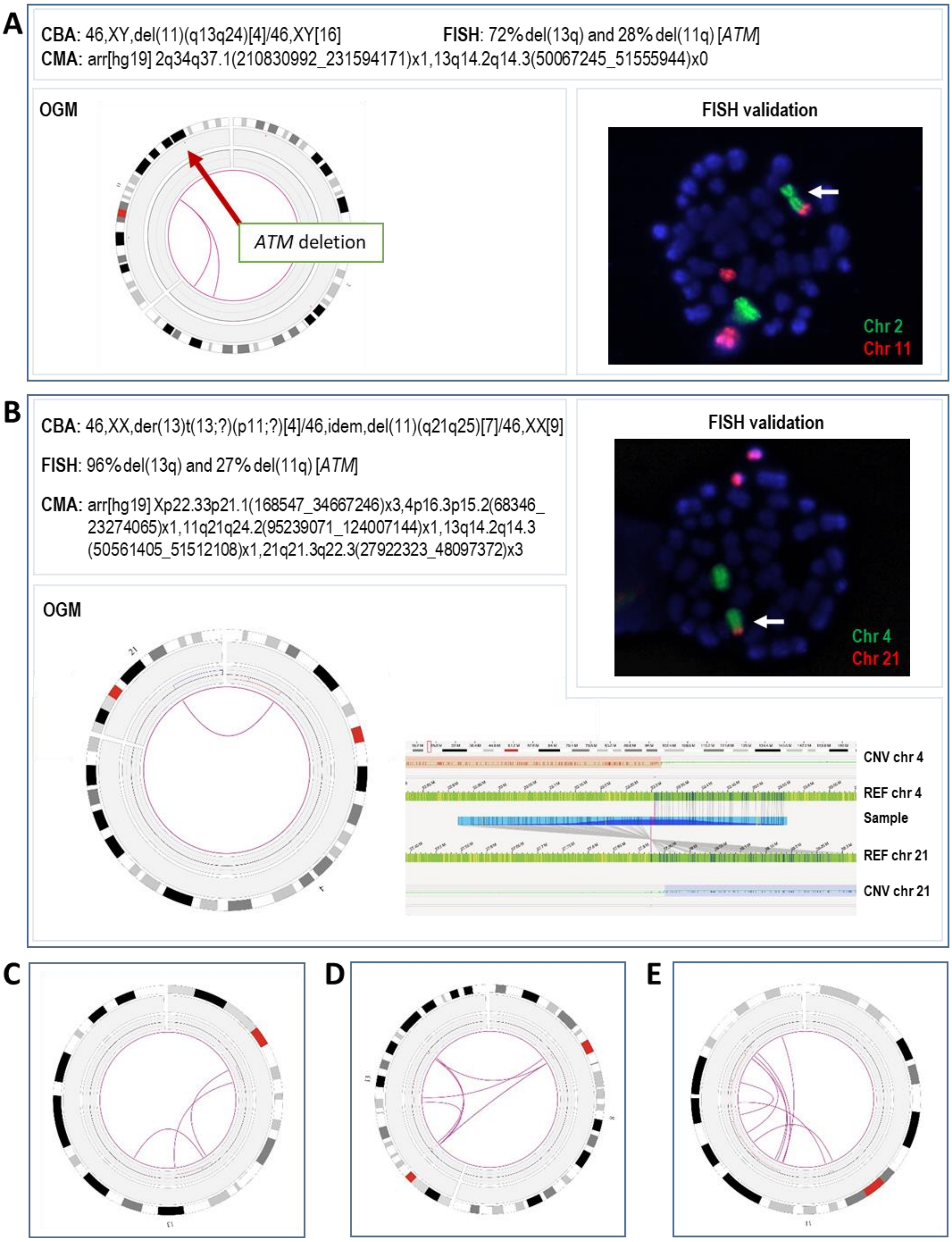

Figure S2. Examples of novel translocations called by OGM that were associated with known CNV previously identified by CMA. (**A**) Patient #11 showed a deletion 11q affecting bands q13q24 as a single abnormality by CBA, also detected in 28% of nuclei by FISH with the 11q22 probe included in the CLL FISH panel. CMA failed to detect this deletion but revealed the presence of a large 20Mb deletion affecting 2q arm missed in the karyotype. Circos plot shows the abnormalities found by OGM on chromosomes 2 and 11, which suggested that the 11q deletion identified was smaller than assumed by conventional techniques (188Kb, including *ATM*) and that the abnormal chromosome 11 detected in the karyotype was a derivative chromosome generated from a cryptic unbalanced t(2;11). Whole-chromosome FISH painting confirmed OGM translocations; (**B**) OGM analysis from patient #12 showed a novel translocation t(4;21) with breakpoints at the limit of two large terminal CNV previously identified by CMA (23Mb loss at 4p and 20Mb gain at 21q). The

rearrangement was confirmed by FISH, which revealed the presence of an unbalanced translocation that was cryptic by CBA; Circos plots showing novel OGM translocations involving known 13q deletions detected by CMA in patient CK5 (**C**) and patient CK9 (**D**), and multiple translocations revealing the high complexity associated with a known *ATM* deletion previously underestimated in patient CK3 (**E**).

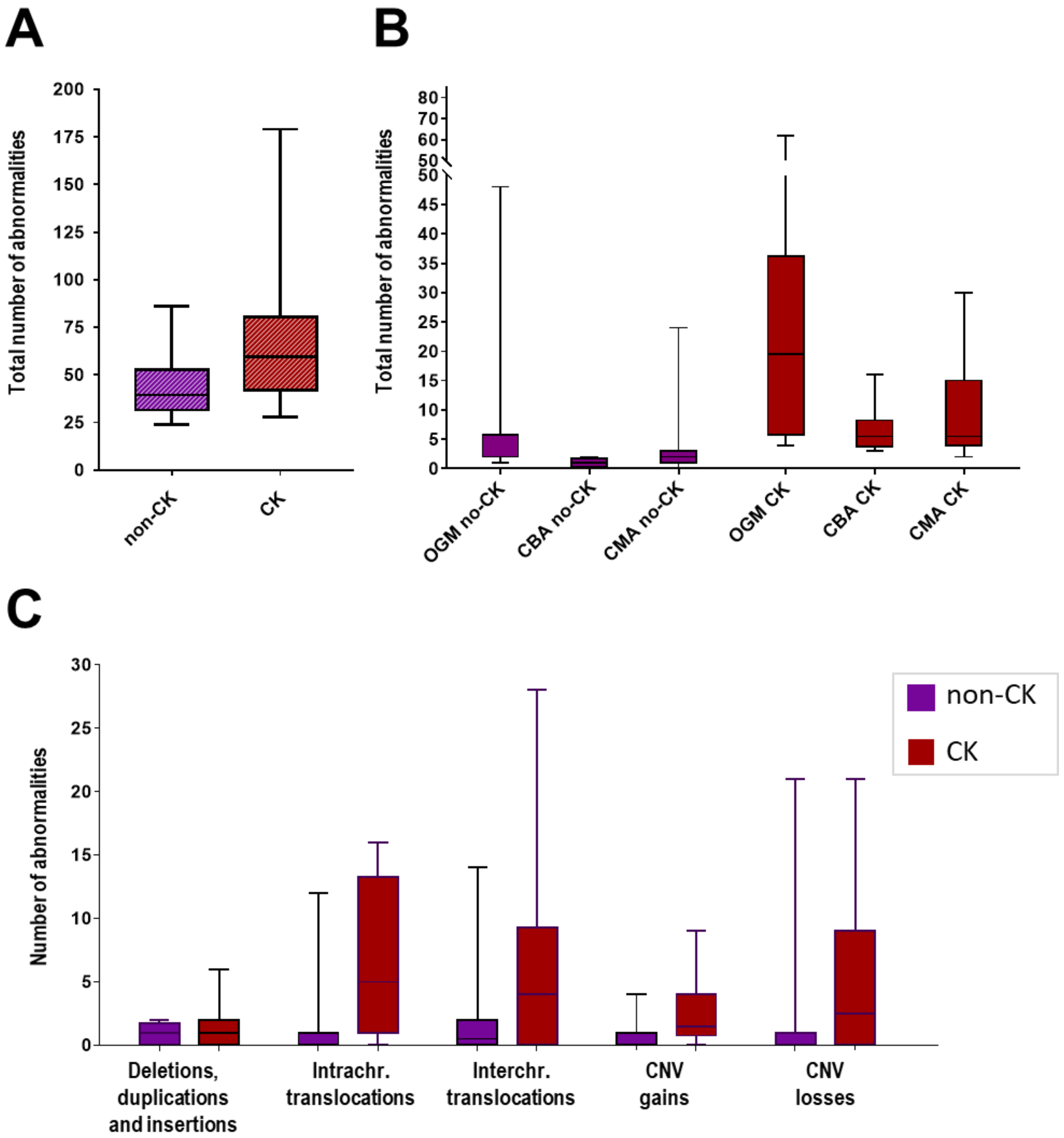

Figure S3. Distribution of the number of abnormalities detected in non-CK and CK groups. (**A**) Total number of SV and CNV called by OGM pipelines; (**B**) Total number of abnormalities by OGM (curated results), chromosome banding analysis (CBA) and chromosomal microarrays (CMA); (**C**) Distribution of the number of the different types of SV and CNV detected by OGM (curated results).

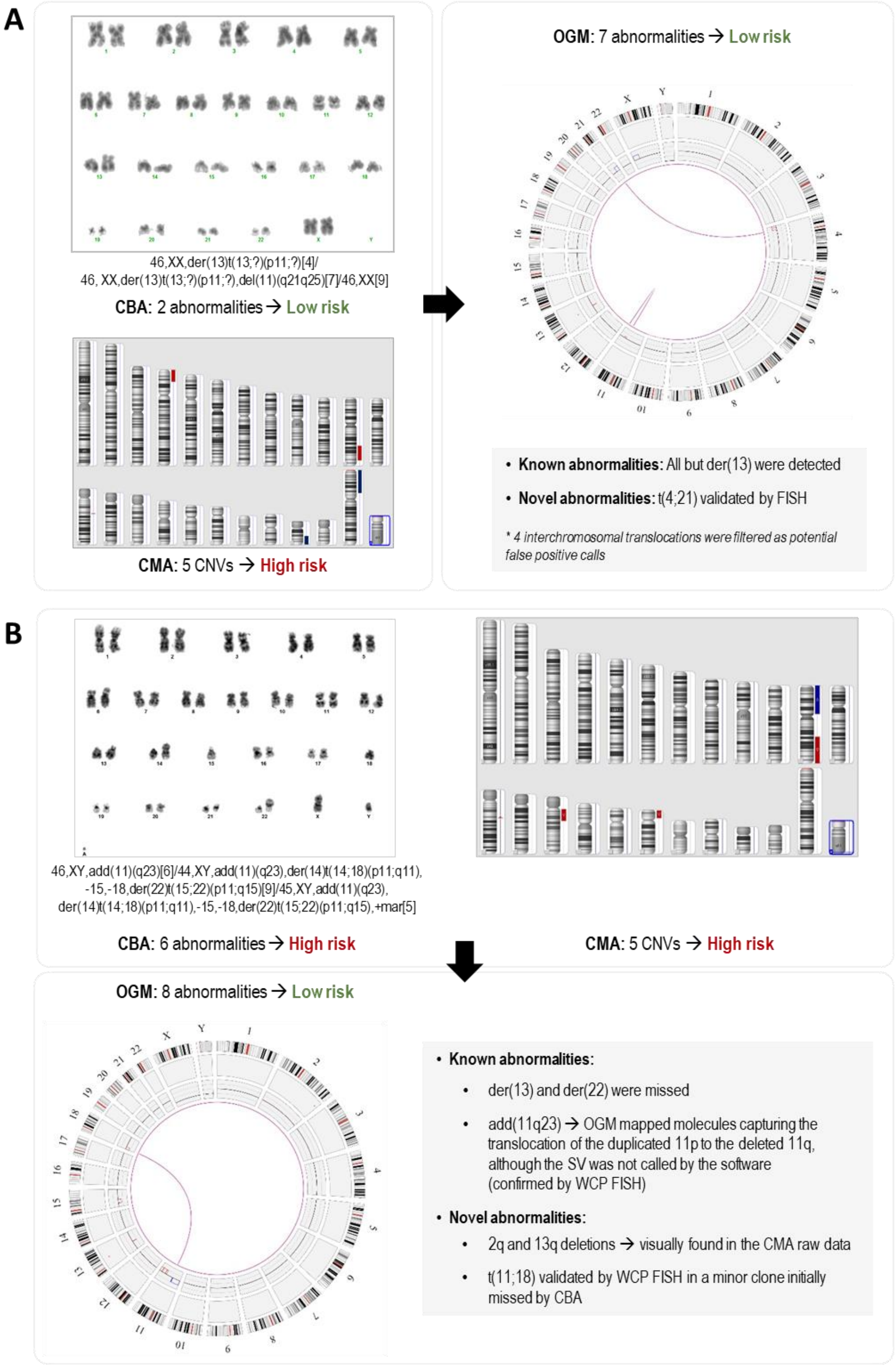

Figure S4. Example of two of the high-risk patients by CBA and/or CMA with less than 10 abnormalities detected by OGM. Detailed results from patient #12 (**A**) and #CK16 (**B**), who were classified in the low complexity group by OGM despite being classified in the highest risk group by at least one of the standard techniques. For each patient karyotype defined by CBA, karyogram gen-

erated by CMA and circos plots showing the curated results obtained by OGM are shown. In addition, the number of abnormalities recorded by each technique and a brief explanation of the comparison among techniques are also detailed.

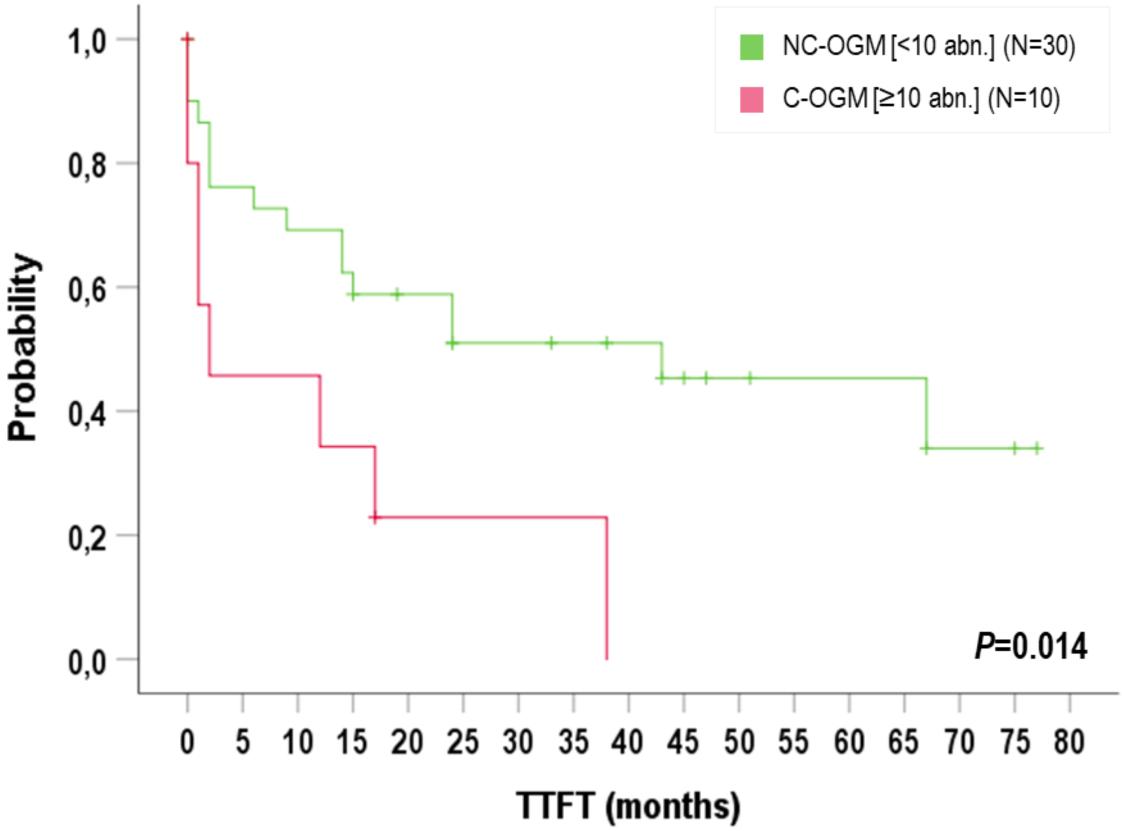

Figure S5. Kaplan-Meier plots for time to first treatment (TTFT) based on the presence of genomic complexity detected by OGM. Patients were divided in two categories based on OGM results: NC-OGM (Non-complex by OGM, <10 abnormalities) and C-OGM (Complex by OGM, ≥10 abnormalities).
